# Evaluating gene by sex and age interactions on cardiovascular risk factors in Brazilian families

**DOI:** 10.1186/1471-2350-11-132

**Published:** 2010-09-20

**Authors:** Suely R Giolo, Alexandre C Pereira, Mariza de Andrade, José E Krieger, Júlia P Soler

**Affiliations:** 1 Laboratory of Genetics and Molecular Cardiology, Heart Institute, Medical School of University of Sao Paulo, Sao Paulo, SP, Brazil; 2 Department of Statistics, Federal University of Parana, Curitiba, PR, Brazil; 3 Department of Health Sciences Research, Mayo Clinic, Rochester, MN, USA; 4 Department of Statistics, University of Sao Paulo, Sao Paulo, SP, Brazil

## Abstract

**Background:**

In family studies, it is important to evaluate the impact of genes and environmental factors on traits of interest. In particular, the relative influences of both genes and the environment may vary in different strata of the population of interest, such as young and old individuals, or males and females.

**Methods:**

In this paper, extensions of the variance components model are used to evaluate heterogeneity in the genetic and environmental variance components due to the effects of sex and age (the cutoff between young and old was 43 yrs). The data analyzed were from 81 Brazilian families (1,675 individuals) of the Baependi Family Heart Study.

**Results:**

The models allowing for heterogeneity of variance components by sex suggest that genetic and environmental variances are not different in males and females for diastolic blood pressure, LDL-cholesterol, and HDL-cholesterol, independent of the covariates included in the models. However, for systolic blood pressure, fasting glucose and triglycerides, the evidence for heterogeneity was dependent on the covariates in the model. For instance, in the presence of sex and age covariates, heterogeneity in the genetic variance component was suggested for fasting glucose. But, for systolic blood pressure, there was no evidence of heterogeneity in any of the two variance components. Except for the LDL-cholesterol, models allowing for heterogeneity by age provide evidence of heterogeneity in genetic variance for triglycerides and systolic and diastolic blood pressure. There was evidence of heterogeneity in environmental variance in fasting glucose and HDL-cholesterol.

**Conclusions:**

Our results suggest that heterogeneity in trait variances should not be ignored in the design and analyses of gene-finding studies involving these traits, as it may generate additional information about gene effects, and allow the investigation of more sophisticated models such as the model including sex-specific oligogenic variance components.

## Background

Despite decades of research, the identification of the genetic basis of complex traits remains a challenging assignment, given that these traits can be influenced by a multiplicity of genetic and environmental factors in that each factor is expected to make a small contribution to trait variability. Regarding complex traits, family studies have proven to be useful in the study of the overall contribution of genes to trait variation and in the comparison of heritability between subgroups such as males and females or young and old individuals. In the comparison of heritability by sex, an indication of gene by sex interaction is provided when sex differences in such heritability are observed.

Several studies have investigated the possibility of heterogeneity in genetic effects by age and sex. In a cohort of Sardinians [1], comparisons of heritability by sex and age (cutoff of 42 yrs between young and old individuals) were performed on many cardiovascular and personality traits with sex and age differences in heritability observed for several traits. For diabetes, hypertension, dyslipidemia and abdominal obesity, part of the operational definition of metabolic syndrome, and also for many other traits, sex differences in heritabilities have also been evaluated and described in numerous other studies [2-5]. In general, results from these studies suggest the effect of a gene by sex interaction for some of the traits that were investigated.

The understanding of the relative importance of both genes and environmental factors for the inter-individual variability of complex traits are paramount for more efficient planning and analysis of mapping and genetic association studies of complex diseases. In fact, the delimitation of a particular stratum of the population, in which the participation of genetic variables has a major importance, may facilitate the identification of small gene effects that may be central to the complex disease causation paradigm.

In this paper, we used the information of 81 families (1,675 individuals) ascertained from a Brazilian family study [6] of cardiovascular risk factors to investigate the heterogeneity in the genetic and environmental contributions to variations in six quantitative cardiovascular risk factors (systolic and diastolic blood pressure, LDL- and HDL-cholesterol, fasting glucose, and triglycerides) by comparing variance components between males and females, and between young and old people. In order to compare our findings with those obtained by [1] we considered the sample median age of 43 years as the cutoff between young and old individuals.

## Methods

### Data set

The data analyzed here are from 81 families of the Baependi Family Heart Study [6]. The data were collected in accordance with a planned sample design from December 2005 to January 2006 in the rural village of Baependi (18,072 inhabitants) located in the state of Minas Gerais, Brazil. All participants provided written informed consent for the collection of samples and subsequent analysis. The study included several stages and was approved by the ethics committee of the Hospital das Clinicas, University of Sao Paulo, Brazil. At the first stage, eleven census districts were selected from among the twelve geographical divisions of Baependi. Next, residential addresses were randomly selected within the districts identified. An individual 18 years or older within each selected household was interviewed, following which all his/her first, second and third degree relatives and his/her respective spouse's relatives that were at least 18 years old were invited to participate in the study. A total of 1,672 individuals from 119 families were interviewed for this study. Each participant provided information regarding family relationships, demographic characteristics, medical history and environmental risk factors. Trained medical students also performed a physical examination and electrocardiogram for each participant. Standard techniques were used for measuring height, weight, blood pressure, fasting blood glucose, total cholesterol, lipoprotein fractions, and triglycerides. Mean values of systolic and diastolic blood pressures were calculated from three measurements taken at three minute intervals.

Considering that families with only one or two individuals cannot provide much information for family studies, we analyzed data from 81 families involving 1,675 people, 43.5% of whom were male. The maximum number of generations per family was four, and the minimum was two (54% of the families had three generations, and 45% had two). In addition, 630 nuclear families were observed as well as 3106 parent-offspring relationships and 2552 grandparent-grandchild relationships. Family size varied from three to 157 individuals, with the average being 21 per family. About 4% of individuals were on lipid-lowering medications, 4% were on hypoglycemic medications, and 24% were on anti-hypertensive medications. Obesity and being overweight were predominant among women due to their sedentary lifestyles. Several descriptive statistics for the individuals analyzed are shown in Table 1.

**Table 1:** Descriptive statistics of 1,675 individuals from 81 Brazilian families

	Total (N = 1675)	Men (N = 729)	Women (N = 946)
Age (years)	44.0 (16.9)	44.5 (17.5)	43.6 (16.5)
BMI (kg/m^2^)	24.4 (4.80)	23.4 (3.80)	25.2 (5.30)
Fasting glucose (mg/dL)	93.7 (30.0)	93.1 (26.5)	94.2 (32.0)
Triglycerides (mg/dL)	133.3 (75.4)	134.8 (82.6)	132.2 (69.3)
HDL-c (mg/dL)	55.9 (15.6)	53.9 (15.3)	57.5 (15.7)
LDL-c (mg/dL)	98.7 (44.1)	96.0 (43.8)	100.7 (44.2)
Systolic blood pressure (mmHg)	126.8 (19.4)	130.3 (18.7)	124.1 (19.4)
Diastolic blood pressure (mmHg)	78.7 (11.4)	79.4 (11.6)	78.3 (11.2)
Smokers (%)	16.6	20.6	13.5
Sedentary lifestyle (%)	21.1	17.8	23.7

Mean values and standard deviations are provided for quantitative traits and % is provided for qualitative traits.

### Statistical Analyses

#### Polygenic model

Consider *y_i _*the measured trait value of the *i*th individual, *i = 1,..., n*, where sets of individuals are grouped into *k *families of *n_f _*relatives (*f = 1,..., k*). Under the polygenic model, *y_i _*is expressed as

(1) $$y_{i}=\mu +\sum_{j=1}^{p}\beta_{j}X_{ij}+g_{i}+e_{i}$$

where *μ *is the general mean of the trait and *β*_*j *_is the regression coefficient for covariate *j *which assumes the value *X_ij _*for individual *i*. The remaining components, *g_i _*and *e_i_*, are the residual genetic effect due to the polygenic term and random error component, respectively. The random effects, *g*_*i *_and *e*_*i*_, are usually assumed to be uncorrelated and normally distributed with means of zero and variance $\sigma_{g}^{2}$ and $\sigma_{e}^{2}$, respectively. The environmental variance component, $\sigma_{e}^{2}$, is unique to each individual. Whereas the polygenic component, $\sigma_{g}^{2}$, is shared between individuals in proportion to their kinship coefficient. Thus, the covariance between traits for individuals *i *and *i' *is given by:

Cov(yi,yi')={σg2+ σ e2     for  i=i'2ϕii'σg2 for i≠i' and related0  for i≠i' and unrelated.

Parameter 2*ϕ*_*ii' *_is the coefficient of relationship between individuals *i *and *i' *The likelihood of the traits of family members is assumed to follow a multivariate normal distribution. Estimates of the mean and variance components are obtained by using maximum likelihood methods [7,8].

In order to test the hypotheses of polygenic effect, H_0_: $\sigma_{g}^{2}$ = 0 against H_0_: $\sigma_{g}^{2}$ > 0, the likelihood ratio statistic can be used. This statistic is asymptotically distributed as a (1/2):(1/2) mixture of $\chi_{1}^{2}$ and $\chi_{0}^{2}$[9]. Under the polygenic model (1) heritability estimates ($h_{g}^{2}$) due to the polygenic effect can be calculated as the proportion of the total variance of the phenotype explained by additive genetic effects after accounting for covariates, i.e. $h_{g}^{2}=\sigma_{g}^{2}/(\sigma_{g}^{2}+\sigma_{e}^{2})$.

#### Models with heterogeneity between the sexes

To model heterogeneity between subgroups of individuals such as males and females, we can evaluate models with separate variance components. Evidence of heterogeneity by sex suggests that models allowing for these differences will be useful in mapping quantitative traits [1]. To evaluate the evidence for heterogeneity in genetic and environmental sources of variation in males and females, we assumed in the polygenic model (1) that $g_{i}\sim \text{N}(0,{\text{ 1}}_{i}\sigma_{g,female}^{2}+\left(\text{1}-{\text{1}}_{i}\right)\sigma_{g,male}^{2})$ and $e_{i}\sim \text{N}(0,{\text{ 1}}_{i}\sigma_{e,female}^{2}+\left(\text{1}-{\text{1}}_{i}\right)\sigma_{e,male}^{2})$, where 1*_i _*is equal to 1 if the individual *i *is female and 0 otherwise. Thus, the covariance between traits for individuals *i *and *i' *is expressed as

$$Cov\left(y_{i},y_{i'}\right)=\{\begin{array}{ll} \sigma_{g,female}^{2}+\sigma_{e,female}^{2} & i=i'\text{ and female }\\ \sigma_{g,male}^{2}+\sigma_{e,male}^{2} & i=i'\text{ and male}\\ 2\phi_{ii'}\sigma_{g,female}^{2} & i\neq i'\text{, related and females}\\ 2\phi_{ii'}\sigma_{g,male}^{2} & i\neq i'\text{, related and males}\\ 2\phi_{ii'}\sigma_{g,female,male}^{2} & i\neq i'\text{, related and }\neq \text{ sexes}\\ \text{0} & i\neq i'\text{ and unrelated}\text{.} \end{array}$$

When individuals *i *and *i' *are related and of opposite sexes, two possible models were evaluated. In the first we assumed that the same set of genes, but with different effects, influence phenotypes for males and females. In this case, the covariance for individuals of opposite sexes was set as *Cov*(*y*_*i*_, *y*_*i'*_) = 2*ϕ*_*ii'*_*σ*_*g,female*_*σ*_*g,male*_. In the second, we assumed that different sets of genes influence phenotypes for males and females. In this case, the covariance was set as *Cov*(*y*_*i*_, *y*_*i'*_) = 2*ϕ*_*ii'*_*ρ*_*sex*_*σ*_*g,female *_with -1 <*ρ*_*sex *_< 1.

Considering the situation where *ρ*_*sex *_= 1, models for each cardiovascular trait were fitted taking into account (a) no covariates, (b) only the sex covariate, and (c) the sex and age covariates, simultaneously. For each of these three situations, four possibilities were considered with regard to the genetic and environmental variance components (v.c.):

1) homogeneity in both of v.c.$\left(\sigma_{g,female}^{2}=\sigma_{g,male}^{2}=\sigma_{g}^{2}\text{ and }\sigma_{e,female}^{2}=\sigma_{e,male}^{2}=\sigma_{e}^{2}\right)$,

2) heterogeneity in at least one of the v.c.$\left(\sigma_{g,female}^{2}\neq \sigma_{g,male}^{2}\text{ and/or }\sigma_{e,female}^{2}\neq \sigma_{e,male}^{2}\right)$,

3) heterogeneity only in the environmental v.c.$\left(\sigma_{g,female}^{2}=\sigma_{g,male}^{2}=\sigma_{g}^{2}\text{ and }\sigma_{e,female}^{2}\neq \sigma_{e,male}^{2}\right)$,

4) heterogeneity only in the genetic v.c.$\left(\sigma_{g,female}^{2}\neq \sigma_{g,male}^{2}\text{ and }\sigma_{e,female}^{2}=\sigma_{e,male}^{2}=\sigma_{e}^{2}\right)$.

Comparison and selection of the best model fitted with no covariates was performed, using tests based on the likelihood ratio statistic. Initially, the hypothesis H_1_: homogeneity in both variance components was tested against H_2_: heterogeneity in at least one of the variance components. In case of rejection of H_1_, conclusion about which variance component is heterogeneous (genetic, environmental or both) was taken after to test the hypothesis a) H_1 _against H_3_: heterogeneity only in the environmental variance component; and b) H_1 _against H_4_: heterogeneity only in the genetic variance component. The same strategy was used to compare and select the best model, including i) the sex covariate, and ii) sex and age covariates, simultaneously. The significance level assumed in this comparison and selection procedure was 0.05.

#### Models with heterogeneity between young and old individuals

To evaluate heterogeneity in variance components by age, we classified individuals into two groups according to the median age in our sample (43 yrs). The younger group included individuals less than or equal to 43 yrs old, and the older group included those above 43 yrs of age. Similar to the model with heterogeneity between the sexes, we assumed in the polygenic model (1) that $g_{i}\sim \text{N}(0,1_{i}\sigma_{g,old}^{2}+(1-1_{i})\sigma_{g,young}^{2})$ and $e_{i}\sim \text{N}(0,1_{i}\sigma_{e,old}^{2}+(1-1_{i})\sigma_{e,young}^{2})$, where 1*_i _*is equal to 1 if the individual *i *is in the older group and 0 otherwise. The covariance between traits for individuals *i *and *i' *is expressed in a similar way as previously described for sex. In this case, the fitted models included (a) no covariates, (b) the age covariate only, and (c) the sex and age covariates, simultaneously. Regarding the genetic and environmental variance components, the possibilities considered were similar to those described for sex with $\sigma_{g,female}^{2}\;,\;\sigma_{g,male}^{2},\sigma_{e,female}^{2}$ and $\sigma_{e,male}^{2}$ replaced by $\sigma_{g,old}^{2},\sigma_{g,young}^{2},\sigma_{e,old}^{2}$ and $\sigma_{e,young}^{2}$ respectively. Models allowing different sets of genes that influence phenotypes in young and old individuals were also investigated. A comparison and selection of the best model was performed in the same manner described for sex.

## Results

### Descriptive statistics

**Figure 1:**
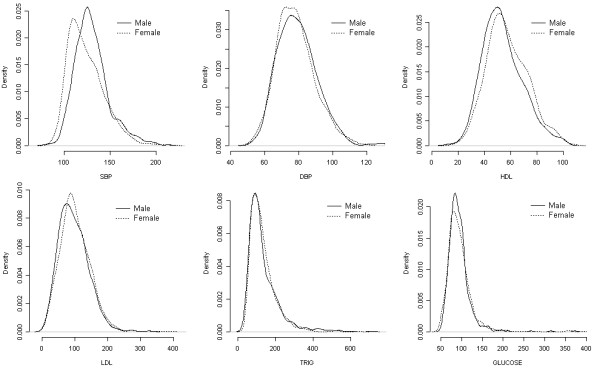
**Distribution of six cardiovascular traits for males and females**. Empirical densities are plotted for males (solid lines) and females (dashed lines) for systolic blood pressure (SBP), diastolic blood pressure (DPB), HDL-cholesterol, LDL-cholesterol, triglycerides (TRG), and fasting blood glucose.

**Figure 2:**
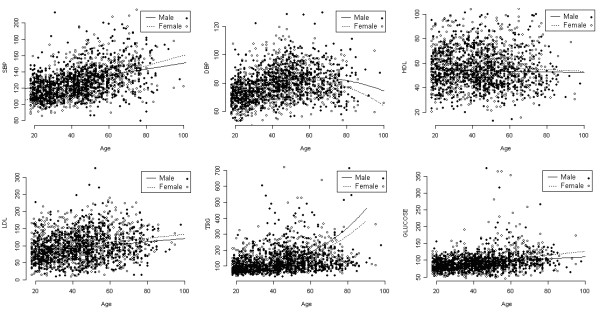
**Values of six cardiovascular traits plotted for males (closed circles) and females (open circles) as a function of age in years**. Regression curves fitted to the data show trends for males (solid lines) and females (dashed lines) with increasing age.

To examine the effect of sex and age on each trait, we generated summary plots for the six traits considered in this paper without considering family structure. Figure 1 displays the distribution of these traits for males and females. From this figure, it is clear that there are no marked differences between the sexes regarding their overall distribution for all traits with the exception of systolic blood pressure. Figure 2 illustrates the effect of age on the same six traits. Regression curves are presented to summarize the effect of age on the traits. Linear trends are observed for four traits (systolic blood pressure, HDL- and LDL-cholesterol, and fasting glucose).

**Figure 3:**
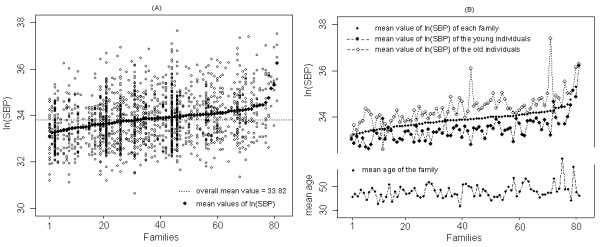
**Values and mean values of ln(SBP) plotted for the Brazilian families**. (A) Values of ln(SBP) plotted for the 81 families where the families are placed in order of their respective mean values. The horizontal dashed line represents the overall mean value of the families. (B) Mean values of ln(SBP) plotted for each family and for young and old individuals within each family, and the mean age profile of the families.

In addition, Figure 3 displays a simple graphic representation [10] that sometimes can be useful for exploring possible heterogeneity in the genetic variance. From graph (A), where the values of the trait ln(SBP) are plotted for each family ordered according to their respective trait means, we note that the mean values associated with the families vary from the overall mean value (horizontal dashed line). This suggests the prevalence of between-family heterogeneity and within-family correlations because, in general, the responses for a family tend to lie on the same side of the overall mean value. From graph (B), it is also possible to see that this pattern of heterogeneity remains, for instance, even after conditioning on young and old age groups. We note that the mean values for individuals in the older group are in general, above the overall mean value. The reverse can be observed for individuals in the younger group. In addition, such a trend is not associated with the mean age profile of the families as displayed in graph (B).

Table 2 displays the mean values and standard deviations of all cardiovascular traits for the entire sample, and for the sample stratified by age and sex simultaneously. The sample median age (43 yrs) was used to classify by age. As expected, trait mean values are, in general, higher for individuals in the older group (> 43 yrs old). Except for HDL-cholesterol, differences in the mean values within sex between age groups were statistically significant at 5%.

**Table 2:** Summary statistics for the entire sample and stratified by sex and age

	Overall		Males (N = 729)				Females (N = 946)			
			Young		Old		Young		Old	
	N = 1675		N = 366		N = 363		N = 489		N = 457	
**Traits**	**Mean**	**s.d**.	**mean**	**s.d**.	**mean**	**s.d**.	**mean**	**s.d**.	**mean**	**s.d**.

SBP (mm Hg)	126.8	19.4	124.8	13.6	135.7	21.5	115.2	14.5	133.5	19.7
DBP (mm Hg)	78.7	11.4	75.8	9.7	82.9	12.3	75.2	10.5	81.6	10.8
HDL (mg/dL)	55.9	15.6	54.6	14.9	53.2	15.5 **	58.3	15.3	56.5	16.0 **
LDL (mg/dL)	98.7	44.1	87.7	39.0	104.5	46.7	93.7	39.6	108.1	47.5
TRG (mg/dL)	133.3	75.4	118.4	73.9	151.3	87.5	111.7	49.2	153.9	80.2
GLU (mg/dL)	93.7	29.6	87.7	17.1	98.7	32.5	86.6	18.8	102.2	39.9

SBP = systolic blood pressure, DBP = diastolic blood pressure, HDL- and LDL-cholesterol, TRG = triglycerides, GLU = fasting glucose, ≤ 43 yrs = young and > 43 yrs = old. **Except for HDL-cholesterol, differences in the mean values within sex between age groups were statistically significant at 5% according to the t-test.

### Heterogeneity in variance components by sex

Since 24% of subjects were on antihypertensive medication, 4% on lipid lowering medication, and 4% on hypoglycaemic medication, a correction factor was applied to those subjects taking medication. This approach has been shown to be superior to ignoring the treatment or to excluding individuals on therapy [11]. In this paper, individuals treated for hypertension were imputed to have 10 mm Hg higher SBP and 5 mm Hg higher DBP than the observed measurements [12,13]. Similarly, individuals on lipid lowering medication were imputed to have 50 mg/dl higher LDL-cholesterol, 10 mg/dl lower HDL-cholesterol and 30 mg/dl higher triglycerides [14,15]. A fixed increment of 30 mg/dl was added to the glucose measurements of those individuals on hypoglycaemic medication [16,17].

After correcting the observed measurements of those subjects taking medication, a series of models was fitted for all traits where heterogeneity in males and females was allowed in polygenic variance component only, in environmental variance component only, and in both variance components. The models were fitted by considering no covariates, the sex covariate only, and sex and age covariates, simultaneously. Although, for DBP and triglycerides, it was suggested (Figure 2) that age^2 ^should be considered in the models, no significant evidence was found to retain it or the interaction between age and sex in the models. Natural log-transformation was applied for all traits in order to have the required normality assumption achieved. For traits where even after transformations the residual kurtosis remained too high, we tried to prevent biased heritability estimates using a robust estimation implemented in SOLAR through the *t-dist *procedure [18]. Under this procedure, the *t*-Student distribution is used in the place of the normal distribution. From fitted models with *ρ*_*sex *_≠ 1 (not shown), we found no evidence that different sets of genes contribute to genetic variation in males and females. Thus, the results shown in Table 3 are only for the models assuming that the same set of genes influence phenotypes for males and females, i.e. *ρ*_sex _= 1, and selected by using the comparison procedure based on likelihood ratio tests described previously.

**Table 3:** Summary of the results obtained for models selected in the analysis of heterogeneity in variance components by sex

		No Heterogeneity				Heterogeneity in Variance Components by Sex					
				
Traits	Covariates	Polygenic heritability estimates	Estimates of polygenic and environmental variances		Selected model	Polygenic heritability estimates		Estimates of polygenic and environmental variances			
						Male	Female	Male		Female	
		**h**^**2**^**(sd)**	${\overset{\wedge }{\sigma }}_{g}^{2}$	${\overset{\wedge }{\sigma }}_{e}^{2}$	variance with heterogeneity	${\text{h}}_{\text{m}}^{2}$	${\text{h}}_{\text{f}}^{2}$	${\overset{\wedge }{\sigma }}_{g,m}^{2}$	${\overset{\wedge }{\sigma }}_{e,m}^{2}$	${\overset{\wedge }{\sigma }}_{g,f}^{2}$	${\overset{\wedge }{\sigma }}_{e,f}^{2}$
**ln(SBP)**	**No** **Sex** **Sex, Age**	0.1412 (0.034) 0.1442 (0.034) 0.2685 (0.041)	0.1783 0.1790 0.2499	1.0841 1.0622 0.6808	environmental environmental no heterogeneity	0.1678 0.1707 0.2685	0.1328 0.1347 0.2685	0.1847 0.1843 0.2499	0.9158 0.8954 0.6808	0.1847 0.1843 0.2499	1.2052 1.1834 0.6808

**ln(DBP)**	**No** **Sex** **Sex, Age**	0.1484 (0.036) 0.1485 (0.036) 0.2305 (0.041)	0.1645 0.1645 0.2241	0.9436 0.8432 0.7479	no heterogeneity no heterogeneity no heterogeneity	0.1484 0.1485 0.2305	0.1484 0.1485 0.2305	0.1645 0.1645 0.2241	0.9436 0.8432 0.7479	0.1645 0.1645 0.2241	0.9436 0.8432 0.7479

**ln(GLU)**	Δ **No** Δ **Sex** Δ **Sex, Age**	0.3401 (0.046) 0.3401 (0.046) 0.3570 (0.048)	0.3250 0.3250 0.3136	0.6305 0.6305 0.5648	both both genetic	0.3184 0.3181 0.2865	0.3520 0.3522 0.4142	0.2635 0.2632 0.2251	0.5640 0.5642 0.5605	0.3744 0.3747 0.3964	0.6891 0.6891 0.5605

**ln(LDL)**	Δ **No** Δ **Sex** Δ **Sex, Age**	0.2956 (0.049) 0.2946 (0.049) 0.2966 (0.050)	0.2903 0.2865 0.2751	0.6918 0.6860 0.6522	no heterogeneity no heterogeneity no heterogeneity	0.2956 0.2946 0.2966	0.2956 0.2946 0.2966	0.2903 0.2865 0.2751	0.6918 0.6860 0.6522	0.2903 0.2865 0.2751	0.6918 0.6860 0.6522

**ln(HDL)**	Δ **No** Δ **Sex** Δ **Sex, Age**	0.3209 (0.047) 0.3207 (0.048) 0.3323 (0.049)	0.4134 0.4096 0.4207	0.8747 0.8674 0.8452	no heterogeneity no heterogeneity no heterogeneity	0.3209 0.3207 0.3323	0.3209 0.3207 0.3323	0.4134 0.4096 0.4207	0.8747 0.8674 0.8452	0.4134 0.4096 0.4207	0.8747 0.8674 0.8452

**ln(TRG)**	**No** **Sex** **Sex, Age**	0.2590 (0.044) 0.2585 (0.044) 0.2863 (0.047)	0.2338 0.2333 0.2328	0.6687 0.6690 0.5803	both both environmental	0.2662 0.2653 0.2507	0.2563 0.2560 0.3324	0.2694 0.2684 0.2370	0.7425 0.7430 0.7082	0.2097 0.2095 0.2370	0.6085 0.6087 0.4759

SBP = systolic blood pressure, DBP = diastolic blood pressure, GLU = fasting glucose, LDL = LDL-cholesterol, HDL = HDL-cholesterol. TRG = triglycerides, Δ = the t-dist procedure used in SOLAR, ${\overset{\wedge }{\sigma }}_{g}^{2}$ and ${\overset{\wedge }{\sigma }}_{e}^{2}$ are estimates of polygenic and environmental variances, ${\overset{\wedge }{\sigma }}_{g,m}^{2}$ and ${\overset{\wedge }{\sigma }}_{g,f}^{2}$ are estimates of male and female polygenic variances, ${\overset{\wedge }{\sigma }}_{e,m}^{2}$ and ${\overset{\wedge }{\sigma }}_{e,f}^{2}$ are estimates of male and female environmental variances. Significance level of 5% was used in the comparison and selection procedure of the best model.

From the selected models presented in Table 3, we observed no significant evidence for heterogeneity of the genetic and environmental variance components in males and females for three traits (diastolic blood pressure, LDL and HDL-cholesterol) either in the presence or absence of the covariates considered. For the fasting glucose trait, models with heterogeneity in both genetic and environmental variance components were selected when either no covariates or the sex covariate was included in the model. In the presence of sex and age covariates, a model with heterogeneity only in the genetic variance component was suggested. Thus, heterogeneity in genetic variance components by sex for this trait does not depend on the covariates included in the model. Models for the triglycerides trait including either no covariates or the sex covariate suggested evidence of heterogeneity in both genetic and environmental variances, while models including sex and age covariates, simultaneously, indicated heterogeneity only in the environmental variance. For the systolic blood pressure (SBP) trait, no heterogeneity in either variance was suggested from model that included the sex and age covariates. In addition, models without the age covariate suggested evidence of heterogeneity only in the environmental variance.

Regarding the polygenic heterogeneity due to sex, two scenarios are highlighted from our results. The first shows genetic heterogeneity in the fasting glucose trait that was maintained independent of the adjustment of the phenotypic mean by covariates. The second scenario shows heterogeneity due to sex in the triglycerides trait that is dependent on age in such a way that if the age effect is adjusted in the phenotypic mean, the effect in the genetic variance component disappears.

In the cases where heterogeneity in the genetic variance component was suggested (fasting glucose and triglycerides traits), higher polygenic heritability between females was observed for fasting glucose.

### Heterogeneity in variance components by age groups

With the purpose of comparing our findings, in terms of the heterogeneity pattern, with those obtained in the cohort of Sardinians [1], it was considered, similar to the Sardinia study, the sample median age (43 yrs) as the cutoff between young and old individuals. Our main interest in this comparison relies on the fact that the Brazilian population has experienced a high level of miscegenation over the centuries [19,20] while the Sardinia population constitutes a genetically isolated founder population [1].

Table 4 displays the results obtained for the same six traits (also after correcting the observed measurements of those subjects taking medication) when heterogeneity for individuals in younger and older groups was allowed in variance components. Results are for the selected models assuming that the same genes influence phenotypes for young and old people (*ρ*_age _= 1) since the opposite was not suggested from the fitted models. As before, models were fitted by considering no covariates, the age covariate only, and the sex and age covariates, simultaneously. Except for the LDL-cholesterol trait, the other traits showed evidence for heterogeneity in at least one of the variance components. For three traits (SBP, DBP and triglycerides), evidence of heterogeneity in the genetic variance was suggested independent of the covariates in the model. Higher polygenic heritability between old people was observed for these three traits (SBP, DBP and TRG). Evidence of heterogeneity only in the environmental variance component was suggested for fasting glucose and HDL-cholesterol independent of the covariates in the model.

**Table 4:** Summary of the results obtained for models selected in the analysis of heterogeneity in variance components by age

		No Heterogeneity				Heterogeneity in Variance Components by Groups of Age					
				
Traits	Covariates	Polygenic heritability estimates	Estimates of polygenic and environmental variances		Selected model	Polygenic heritability estimates		Estimates of polygenic and environmental variances			
						Young	Old	Young		Old	
		**h**^**2**^**(s.d.)**	${\overset{\wedge }{\sigma }}_{g}^{2}$	${\overset{\wedge }{\sigma }}_{e}^{2}$	variance with heterogeneity	${\text{h}}_{\text{y}}^{2}$	${\text{h}}_{\text{o}}^{2}$	${\overset{\wedge }{\sigma }}_{g,y}^{2}$	${\overset{\wedge }{\sigma }}_{e,y}^{2}$	${\overset{\wedge }{\sigma }}_{g,o}^{2}$	${\overset{\wedge }{\sigma }}_{e,o}^{2}$
**ln(SBP)**	**No** **Age** **Sex, Age**	0.1412 (0.034) 0.2647 (0.041) 0.2685 (0.041)	0.1783 0.2509 0.2499	1.0841 0.6969 0.6808	genetic genetic genetic	0.0016 0.0099 0.0117	0.1512 0.2008 0.2155	0.0014 0.0065 0.0067	0.8477 0.6468 0.5639	0.1510 0.1625 0.1549	0.8477 0.6468 0.5639

**ln(DBP)**	**No** **Age** **Sex, Age**	0.1484 (0.036) 0.2306 (0.041) 0.2305 (0.041)	0.1645 0.2242 0.2241	0.9436 0.7480 0.7479	genetic genetic genetic	0.0420 0.0252 0.0330	0.1801 0.1625 0.1938	0.0399 0.0244 0.0254	0.9100 0.9440 0.7438	0.1999 0.1832 0.1788	0.9100 0.9440 0.7438

**ln(GLU)**	Δ **No** Δ **Age** Δ **Sex, Age**	0.3401 (0.046) 0.3571 (0.048) 0.3570 (0.048)	0.3250 0.3137 0.3136	0.6305 0.5647 0.5648	environmental environmental environmental	0.9554 0.9324 0.9330	0.5058 0.5093 0.5095	0.3346 0.3163 0.3163	0.0156 0.0229 0.0227	0.3346 0.3163 0.3163	0.3269 0.3047 0.3045

**ln(LDL)**	Δ **No** Δ **Age** Δ **Sex, Age**	0.2956 (0.049) 0.2983 (0.050) 0.2966 (0.050)	0.2903 0.2796 0.2751	0.6918 0.6575 0.6522	no heterogeneity no heterogeneity no heterogeneity	0.2956 0.2983 0.2966	0.2956 0.2983 0.2966	0.2903 0.2796 0.2751	0.6918 0.6575 0.6522	0.2903 0.2796 0.2751	0.6918 0.6575 0.6522

**ln(HDL)**	Δ **No** Δ **Age** Δ **Sex, Age**	0.3209 (0.047) 0.3323 (0.048) 0.3323 (0.049)	0.4134 0.4245 0.4207	0.8747 0.8529 0.8452	environmental environmental environmental	0.4547 0.4467 0.4546	0.3652 0.3729 0.3755	0.4276 0.4357 0.4325	0.5126 0.5395 0.5188	0.4276 0.4357 0.4325	0.7430 0.7327 0.7193

**ln(TRG)**	**No** **Age** **Sex, Age**	0.2590 (0.044) 0.2874 (0.047) 0.2863 (0.047)	0.2338 0.2339 0.2328	0.6687 0.5799 0.5803	genetic genetic genetic	0.0843 0.1263 0.0268	0.2680 0.2385 0.2600	0.0633 0.0826 0.0169	0.6875 0.5714 0.6130	0.2518 0.1790 0.2154	0.6875 0.5714 0.6130

SBP = systolic blood pressure, DBP = diastolic blood pressure, GLU = fasting glucose, LDL = LDL-cholesterol, HDL = HDL-cholesterol. TRG = triglycerides, Δ = the t-dist procedure used in SOLAR, ${\overset{\wedge }{\sigma }}_{g}^{2}$ and ${\overset{\wedge }{\sigma }}_{e}^{2}$ are estimates of polygenic and environmental variances, ${\overset{\wedge }{\sigma }}_{g,y}^{2}$ and ${\overset{\wedge }{\sigma }}_{g,o}^{2}$ are estimates of young and old polygenic variances, ${\overset{\wedge }{\sigma }}_{e,y}^{2}$ and ${\overset{\wedge }{\sigma }}_{e,o}^{2}$ are estimates of young and old environmental variances. Significance level of 5% was used in the selection procedure of the best model.

## Discussion and Conclusions

In the analysis of cardiovascular traits, we used information on 81 Brazilian families (1,675 individuals) to investigate the heterogeneity in variance components in subgroups of individuals. In particular, we investigated such heterogeneity by the two factors most commonly considered in the literature: sex and age. The sex and age dependency in the variances (as well as in the means) of the traits could indicate that different genetic and environmental factors may be influential in different sex-age cohorts [21]. Investigation of gene by sex interactions, for instance, is relevant because it may help to clarify differences on genetic susceptibilities and explain the sexual dimorphism of complex traits [5].

Here, we found evidence of age and/or sex differences in variance components for some of the cardiovascular traits analyzed. When analyzed by age, for instance, such differences affected the polygenic variance for triglycerides, and systolic and diastolic blood pressure, independent of the covariates in the models; but for fasting glucose and HDL-cholesterol, they affected the environmental variance. The age dependency in polygenic variance can be due to different magnitudes of the heritability with age or because different genes affect the trait at different ages [21]. Therefore, heterogeneity in variances should not be ignored in the analyses of these complex traits, even in the absence of genotypic data, since it may provide a strong argument for further research with the goal of mapping potentially different genes involved in males and females, as well as according to age. When genotypic data are available, more sophisticated models can be investigated for finding genes, such as the model including sex-specific oligogenic variance components (i.e. sex-specific components associated with major genes or QTL's). In an effort to illustrate the polygenic effect and its possible heterogeneity in subgroups of individuals as well as the possible covariate effects, we presented simple descriptive graphics in this work that may help in the understanding of the familial variation and correlation, although, in our illustrations, the genetic distances between individuals within families were not taken into account.

A point that can be highlighted from heterogeneity analysis is that care in the interpretation of the polygenic heritability is needed when genetic variance components are suggested to be homogeneous, but not the environmental variance components. In these cases, polygenic heritabilities will be, for instance, different by sex but only in a relative meaning due to environmental influences. In our study, this situation was observed in the evaluation of sex differences in the heritability of triglycerides.

In terms of the heterogeneity pattern, the results that we obtained for the Brazilian families in the Baependi Heart Study were similar to those obtained for the Sardinian families [1]. As in the Sardinian study, it was noted in our findings that two of the most heritable traits in the older group were systolic and diastolic blood pressure (SBP and DBP). The heterogeneity in variances for traits like these can be therefore too great to be ignored. Thus, modeling the variance heterogeneity between groups could be valuable in molecular studies. In such studies it may also be desirable to focus attention on the most informative individuals. For SBP and DBP traits, for instance, a very low heritability was observed for individuals less than 43 years of age. In this case, it could be therefore more fruitful to focus molecular studies on older individuals, since they showed higher heritability.

Overall, both this present study and other similar studies have supported sex and age differences in the polygenic effect on some cardiovascular traits [21]. The importance of identification of gene by sex and/or age interaction has therefore shown to suggest that, in the new genome era, it may contribute to the development of sex- and age-specific preventive and therapeutic strategies. The study of interactions, however, should not be restricted to the influence of sex and age groups. Other factors, e.g. race, should also be interesting to consider in the study of complex traits to increase the power of the analysis beyond pleiotropic influences [22].

In this paper we used the cutoff of 43 years between young and old. However, depending on the focus of the analysis, other cutoffs may be of greatest interest, since some of the sex-specific variation identified in serum lipids and other metabolic traits are tied to serum levels of sex hormones levels. Probably, and as has been recently proposed [23], the influence of continuous factors like age, commonly considered in groups, could provide more interesting information if considered as continuous in the study of gene by age interaction of complex traits. For this purpose, however, additional methodological and computational efforts may be needed.

## Competing interests

The authors declare that they have no competing interests.

## Authors' contributions

SRG, ACP and JPS performed the statistical analyses. SRG drafted the manuscript under the supervision of JPS, ACP, MA and JEK. ACP, JPS and JEK supervised the study. All authors read and approved the final manuscript.

## Pre-publication history

The pre-publication history for this paper can be accessed here:

http://www.biomedcentral.com/1471-2350/11/132/prepub
